# Retraction: Exploring novel fluorine-rich fuberidazole derivatives as hypoxic cancer inhibitors: Design, synthesis, pharmacokinetics, molecular docking, and DFT evaluations

**DOI:** 10.1371/journal.pone.0348537

**Published:** 2026-05-05

**Authors:** The PLOS One Editors

After this article [1] was published, concerns were raised regarding the results presented in S1 File and with the statistical analysis.

Specifically:

- In S1 File in [1], similarities were noted in the baseline of the NMR spectra between Figs S6 and S8 and between Figs S26 and S28, despite these spectra representing different compounds.
- The Nonlinear optical and optoelectronic studies subsection of the Materials and methods section reports that data were analyzed using Student’s t-test; however:
  - multiple experiments in this article [1] appear to present more than two conditions
  - the Results and discussion section, tables, and figures do not appear to report p values.

Regarding the NMR spectra in S1 File, the corresponding author stated the similarities in the baseline noise between Figs S6 and S8 and between Fig S26 and S28 arose from the use of identical instrumental acquisition parameters, standardized processing protocols, and uniform digitization procedures.

The corresponding author stated that statistical analyses were performed for Figs 4 and 5 and they provided the original underlying data files used to plot Figs 4 and 5, and perform the statistical analyses. Editorial review of these files raised additional concerns for the results presented in [1], including:

- Regarding the results presented in Fig 4, the Results and discussion section reports compound **5a** had no discernible effect on H2AX phosphorylation and that compound **5i** results in improved H2AX phosphorylation, however these statements are not supported by the underlying statistical analyses.
- Regarding the results presented in Fig 5, the Results and discussion section states after 24 and 48 hours, no elevation in the activity of caspase 3/7 was observed when hypoxic cells were treated with chemicals (**5a, 5c-5e, 5g-5i,** and **5m**) relative to control, however the underlying statistical analyses showed these compounds significantly decreased caspase 3/7 Luminescence [RLU].
- The error bars in Figs 2-5 in [1] do not appear to match the underlying individual-level data provided in post-publication discussions.

In light of the above unresolved concerns that question the reliability and integrity of the reported results and conclusions, the *PLOS One* Editors retract this article.

MBT responded but expressed neither agreement nor disagreement with the editorial decision. AR, RA, AMA, MA, AHH, WA, SN, SU, AGAS, RS, NAB, and HA either did not respond directly or could not be reached.
